# Time and distance estimation in children using an egocentric navigation task

**DOI:** 10.1038/s41598-018-36234-1

**Published:** 2018-12-20

**Authors:** Kay Thurley, Ulrike Schild

**Affiliations:** 1 Department Biology II, Ludwig-Maximilians-Universität München, Munich, Germany; 2 Bernstein Center for Computational Neuroscience Munich, Munich, Germany; 3 Developmental Psychology, University of Tübingen, Tübingen, Germany

## Abstract

Navigation crucially depends on the capability to estimate time elapsed and distance covered during movement. From adults it is known that magnitude estimation is subject to characteristic biases. Most intriguing is the regression effect (central tendency), whose strength depends on the stimulus distribution (i.e. stimulus range), a second characteristic of magnitude estimation known as range effect. We examined regression and range effects for time and distance estimation in eleven-year-olds and young adults, using an egocentric virtual navigation task. Regression effects were stronger for distance compared to time and depended on stimulus range. These effects were more pronounced in children compared to adults due to a more heterogeneous performance among the children. Few children showed veridical estimations similar to adults; most children, however, performed less accurate displaying stronger regression effects. Our findings suggest that children use magnitude processing strategies similar to adults, but it seems that these are not yet fully developed in all eleven-year-olds and are further refined throughout adolescence.

## Introduction

The question of how the senses of time and space develop from infancy to adulthood has a long tradition in psychology going back to the roots of the famous experiments by Piaget^1,2^. Piaget placed the cognitive cornerstone of time development at ages between seven and eleven years when children produce logical solutions to concrete problems using language. Recently, however, applying implicit testing paradigms like eye-tracking methods, a sense of time has been shown even in infants with striking similarities to adults (e.g. Refs.^3–6^). Similarly, research concerning spatial competence suggests that some spatial skills like coding for location and distances might already be present early in development (see Ref.^7^ p. 9/10 for a review). However, different spatial skills, like object use and reaching in near space, object location, navigation in far space, spatial scaling, and mental imagery^8,9^ may have different developmental trajectories. The development of navigational capabilities has only been examined in few studies. Ribordy *et al*.^10^ have shown that basic allocentric spatial memory abilities already appear around two years of age. Three-year-olds combine egocentric/allocentric strategies for spatial memory; whereas five-year-olds use viewpoint-independent strategies based on landmarks indicating an improvement of the relevant cognitive abilities (incl. visuospatial memory and executive control) in preschool and school age^11,12^. Nevertheless, orientation using landmarks may still be less accurate, showing different error patterns in 5 to 7-year-old children compared to adults^13^. Similarly, the accuracy in using egocentric information for navigation increases from 5 to 7 years of age^14^, but its integration with allocentric cues is not mature in 6- to 11-year-olds^15^. Although core components of spatial and timing competence might be present from infancy on^16^ (but see Ref.^17^ for a critical discussion of the nativist-empirist debate within spatial development), refinements surely take place during childhood^9^. These refinements also include cognitive factors that help solving timing or spatial tasks like understanding of verbal instructions, attentional focus on the task and motor demands^18^, processing speed^19^, and short-term memory^20^. It is therefore sometimes hard to determine whether developmental changes in timing or spatial tasks are simply due to changes in such cognitive factors. Moreover, like in adults^21–23^ experience in music or training in other time-related activities like certain sports, might favor specific strategies in task solving and hence influence performance in children.

The estimation of physical magnitudes is influenced by systematic biases (for a nice overview of the different biases see Ref.^24^). Most intriguing is the *regression effect*, i.e. systematic over- and underestimations for small and large stimuli, respectively, over a testing range. The judgements thus gravitate towards the mean of the stimulus distribution; therefore, the regression effect is also referred to as regression to the mean or central tendency. This effect has been first described for time judgements (as Vierordt’s law^25^) but has been observed for other magnitudes since then^26^ including number^27^, and distance^28^. The regression effect is differently modulated by statistical features of the test range^29^, e.g., the strength of the regression effect increases with the mean of the test range^28,30^. These influences are subsumed under the term *range effect*.

To our knowledge only few studies have investigated regression and range effects in children. For example, Karaminis *et al*.^31^ found a progressive decrease in the strength of the regression effect with age for estimation of ~1 s intervals in children between 6 and 14 years, and adults. Concerning distance estimation, Sciutti *et al*.^32^ investigated the reproduction of lengths between 2 and 14 cm. They asked children from 7 to 14 years and adults to reproduce the distance between two light flashes displayed on a screen by finger movements. Children and adults showed regression effects of similar strength. In contrast, spatial discrimination, which was tested in a second experiment and assessed via Weber ratios, was less precise in younger children and became more accurate with age. Despite testing for two partly overlapping ranges of stimuli, Sciutti *et al*.^32^ only showed visual evidence for range effects but did not analyze their data for range effects.

**Figure 1:**
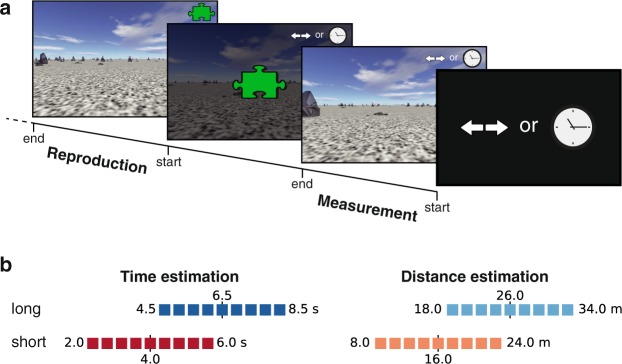
Time and distance reproduction task. (**a**) Sequence of trial events. Each trial started with the presentation of a double arrow or a clock symbol on black background, cueing a distance or time estimation trial, respectively. Only either distance or time stimuli were given in one mission. Upon pressing the space bar of the PC’s keyboard, the display changed to show simulated linear movement through a desert landscape. In the upper right corner, a small version of the symbol was shown, indicating task type. When target distance or time was reached, movement stopped automatically and a green puzzle symbol appeared denoting the start of the reproduction phase. Pressing the space bar another time changed the display to movement through the desert. The participant decided to finish reproduction by releasing the space bar. (**b**) Stimulus distributions. Two different stimulus distributions (short and long ranges) were used for time and distance estimation experiments, respectively. Distributions were uniform and only differed by their means (marked by a vertical black solid line and number). Stimuli were chosen from nine values evenly spaced across each distribution (colored squares). Upper and lower borders are given as numbers. Color identity is kept throughout the article.

In the present study, we investigated regression and range effects for time and distance estimation in eleven-year-old children and adults. To be able to test for both modalities in the same experiment, we used an egocentric navigation task (path integration); cf. Figure 1a. Participants had to move through a computer-generated environment (visual self-motion in virtual reality) and estimate either the duration or the distance of their movement (measurement phase). Immediately thereafter, they had to reproduce the estimated duration or distance, respectively, by moving again through the virtual environment (reproduction phase). Distances were chosen between 8 and 34 m, a reasonable stimulus range for path integration. Time stimuli had matching values between 2 and 8.5 s, thus we tested for supra-second intervals compared to many other timing studies. To disentangle elapsed time and covered distance, we changed the movement speed in every trial. We could therefore also examine the influence of movement speed on time and distance estimation. Finally, we explored how individual differences in cognitive measures (e.g., general attention and subjective attention during the task), daily activities (like doing sports, playing a musical instrument, and playing computer games) and judgements about applied strategies in the task modulate regression and range effects.

## Results

### Time and distance estimation in children

We conducted experiments with 10 to 12-year-old children in which they first estimated and later reproduced either the duration or the distance of visual self-motion through a computer-generated landscape (Fig. 1a). Stimuli in each session were randomly drawn from one of four uniform distributions, comprising duration or distance stimuli and shorter or longer stimulus ranges (Fig. 1b).

**Figure 2:**
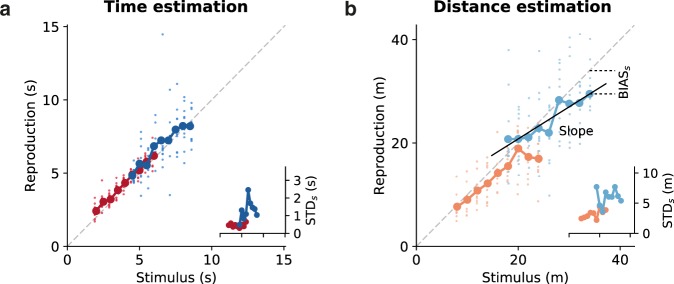
Time and distance reproductions of an example child. Individual reproduced values for stimulus interval (**a**) or distance (**b**) are given as small dots and averages for each stimulus as large dots connected by a solid line. Colors identify stimulus distributions (cf. Fig. 1b). Gray dashed lines mark bisecting lines. The insets give the standard deviation STD_*s*_ for each stimulus. The x-axis is the same as in the main panel. During time estimation reproduction is veridical in this participant. For distance estimation, regression effects are visible in particular for the long stimulus range. An increased standard deviation for larger stimuli can be observed for both time and distance estimation. In (**b**) the bias for the largest stimulus BIAS_*s*_ and a linear fit (black solid line) from which we extracted the slope are illustrated.

Reproduced values depended on stimulus modality and range. Figure 2 gives data of one example child. During time estimation, reproduction was veridical in this participant. For distance estimation, however, regression effects are visible − in particular for the long stimulus range. Standard deviation increased with stimulus size for both time and distance estimation (scalar variability, i.e., the increase of the variability of an estimate with the magnitude of the stimulus; a consequence of the Weber-Fechner law).

**Figure 3:**
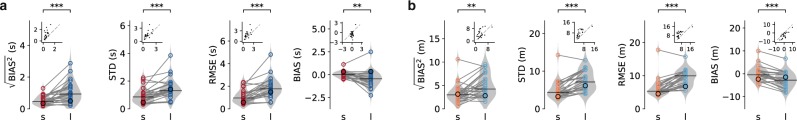
Characteristics of time and distance estimation in children. Comparison of short (s) and long (l) stimulus ranges for $\sqrt{{{\rm{BIAS}}}^{2}}$, standard deviation, RMSE, and BIAS for all 24 children. (**a**) Data for time and (**b**) distance estimation. Data of individuals is displayed as open circles connected by a gray line. Filled circles belong to the example participant from Fig. 2. Violin plots illustrate the distribution of the population. A solid black line marks the median. Stars indicate significant differences (Wilcoxon signed-rank tests) with *p* ≤ 0.05, *p ≤ 0.01, **p ≤ 0.001, ***. Insets: Same data as in the main panel but plotting short vs. long for each participant (black dots). Gray dashed lines mark the bisecting lines.

To quantify the results across all children, we extracted different parameters (see Methods for details). The square-root of the mean squared bias $\sqrt{{{\rm{BIAS}}}^{2}}$ gives an estimate of the deviation between stimulus and reproduction. For time estimation, it was significantly increased with the long stimulus range compared to the short one (Wilcoxon signed-rank test, *W* = 9, *p* = 4 × 10^−6^), suggesting a stronger regression effect for the long stimulus range (Fig. 3a). Similarly, and in accordance with scalar variability, the standard deviation was larger for the long compared to the short range (*W* = 26, *p* = 1 × 10^−4^) as was the RMSE (*W* = 13, *p* = 1 × 10^−5^). Calculating the (signed) BIAS revealed a general underestimation for the long but not the short range (*W* = 249, *p* = 3 × 10^−3^). This partly explained the larger $\sqrt{{{\rm{BIAS}}}^{2}}$.

A similar picture was found for distance estimation, with stronger $\sqrt{{{\rm{BIAS}}}^{2}}$ (*W* = 54, *p* = 4 × 10^−3^), STD (*W* = 6, *p* = 2 × 10^−6^), RMSE (*W* = 8, *p* = 3 × 10^−6^) and BIAS (*W* = 291, *p* = 4 × 10^−6^) for the long compared to the short range (Fig. 3b).

To prevent distance estimation in a timing experiment and vice versa, we randomly adapted the movement speed in each reproduction trial. Since choice was made from discretized stimulus and speed distributions, subsets of trials had matching stimulus configuration (duration, simultaneously covered distance, and speed) in both time and distance estimation experiments. To test for systematic influences of distance covered on time estimation and vice versa, we compared the actual time/distance reproductions with the calculated “time”/“distance” reproductions from their counterpart session. Supplementary Fig. S1a shows the data for the child in Fig. 2. Most remarkable is the considerably larger variance in the calculated reproductions from the counterpart session. Similarly, for all children measures sensitive to variability (STD, RMSE, coefficient of variation) were increased for calculated reproductions (Supplementary Fig. S2). This increase in variability is the consequence of the decoupling of time and distance by trial-by-trial changes in movement speed. In contrast, the slope was not systematically affected across all children. Interestingly, the BIAS (and as a consequence also the $\sqrt{{{\rm{BIAS}}}^{2}}$) increased for calculated time responses from distance sessions. As another test, we correlated matching pairs of actual and calculated reproductions. These correlations were significant for most children but weak due to the decoupling procedure (Supplementary Figs. S4 and S1b for an example).

### Reproduction performance vs. specific skills in children

To explore possible sources of individual differences in time and distance estimation, we correlated our statistical measures with the d2-R^33^ [raw score of concentration performance mean 106.6, STD 15.3, range 76–143] and with questionnaire data on the amount of time spent doing sports (mean 3.4, STD 1.2), playing a musical instrument (mean 2.8, STD 1.6), and playing computer games (mean 2.7, STD 1.3). Further they were correlated with indications whether and how often children used a rhythm (mean 2.4, STD 1.3) and another strategy (mean 1.9, STD 1.3) for task solving, and how much effort they put into performing well (mean 1.7, STD 0.8). We obtained significant correlations for playing a musical instrument and $\sqrt{{{\rm{BIAS}}}^{2}}\,$(*ρ* = −0.45*, p* = 0.027), STD (*ρ* = −0.65*, p* = 6 × 10^−4^) and RMSE (*ρ* = −0.64*, p* = 8 × 10^−4^) of time estimations in the short range, and with STD (*ρ* = −0.50*, p* = 0.013) and RMSE (*ρ* = −0.43*, p* = 0.038) of time estimations in the long range. All correlations were negative. This suggests that children who frequently play a musical instrument had fewer variability in time estimation than children who do not (or seldom do). Applying a strategy correlated with BIAS (*ρ* = −0.46*, p* = 0.023) only for the short range in time estimation. Applying a rhythm correlated with $\sqrt{{{\rm{BIAS}}}^{2}}$ (*ρ* = −0.45*, p* = 0.026) and RMSE (*ρ* = −0.42*, p* = 0.042) for the long range in time estimations (see Supplementary Tab. S1 for all correlations).

### Time and distance estimation in adults

**Figure 4:**
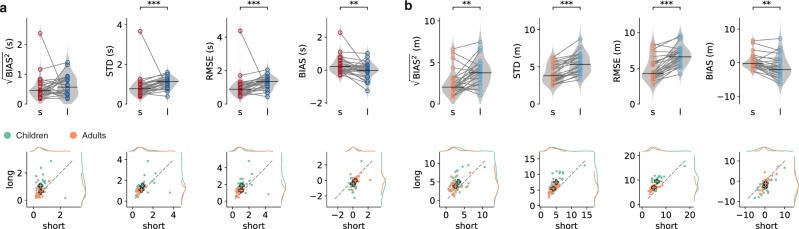
Characteristics of time and distance estimation in adults compared to children. The upper panels display data for all 24 adult participants during time (**a**) and distance estimation (**b**). See caption of Fig. 3 for a description. Lower panels compare adults (orange) to children (green), plotting the values for each participant of the parameter displayed in the above panel for short vs. long ranges. Colored plus signs mark averages for children and adults, respectively. Gray dashed lines are bisecting lines. Corresponding marginal distributions are given at the right and the top.

We repeated the above experiments with 24 young adults aged between 20 and 27 years. As for children, time and distance estimation depended on stimulus range with increased $\sqrt{{{\rm{BIAS}}}^{2}}$ (distance: *W* = 41, *p* = 0.0011; time: *W* = 97, *p* = 0.135 failed to reach significance), STD (time: *W* = 27, *p* = 1 × 10^−4^; distance: *W* = 0, *p* = 1 × 10^−7^), RMSE (time: *W* = 34, *p* = 4 × 10^−4^; distance: *W* = 1, *p* = 2 × 10^−7^), and smaller BIAS for long than for short ranges (time: *W* = 255, *p* = 0.002, with slightly positive medians; distance: *W* = 263, *p* = 6 × 10^−4^); see Fig. 4.

Comparison of the actual reproductions to the ones calculated from counterpart sessions as for children showed an increase in variability and small correlations between matching pairs of actual and calculated reproductions (see Supplementary Figs. S3 and S4). The increase of $\sqrt{{{\rm{BIAS}}}^{2}}$ and BIAS for calculated time from sessions with distance estimation was even more pronounced in adults compared to children, indicating an influence of the passage of time in distance estimation; and possibly vice versa, since the $\sqrt{{{\rm{BIAS}}}^{2}}$ for calculated distance reproductions was smaller for the long times compared to the long distances (Supplementary Fig. S3).

### Comparison of children and adults

We performed two-way rANOVAs to compare children and adults. These analyses revealed more pronounced values in children compared to adults for $\sqrt{{{\rm{BIAS}}}^{2}}$ (*F*_*1,46*_ = 4.73, *p* = 0.035, *η*^2^ = 0.06), BIAS (*F*_*1,46*_ = 5.06, *p* = 0.029, *η*^2^ = 0.07) and RMSE (*F*_*1,46*_ = 4.01, *p* = 0.051, *η*^2^ = 0.06) in time estimation as well as for $\sqrt{{{\rm{BIAS}}}^{2}}$ (*F*_*1,46*_ = 4.67, *p* = 0.036, *η*^2^ = 0.07), STD (*F*_*1,46*_ = 8.94, *p* = 0.004, *η*^2^ = 0.14) and RMSE (*F*_*1,46*_ = 12.05, *p* = 0.001, *η*^2^ = 0.18) in distance estimation (Fig. 4). Range effects were apparent in both children and adults for all parameters, i.e. absolute values were always larger for long ranges in both time and distance experiments (all, *F*_*1,46*_ ≥ 14.83, *p* ≤ 0.001, *η*^2^ ≥ 0.10). In addition, interactions between group and range were obtained for time estimation in $\sqrt{{{\rm{BIAS}}}^{2}}$ (*F*_*1,46*_ = 9.42, *p* = 0.004, *η*^2^ = 0.07) and RMSE (*F*_*1,46*_ = 5.84, *p* = 0.02, *η*^2^ = 0.04) and for distance estimation in STD (*F*_*1,46*_ = 7.77, *p* = 0.008, *η*^2^ = 0.02) and RMSE (*F*_*1,46*_ = 6.42, *p* = 0.015, *η*^2^ = 0.02). These interactions indicate that the difference of values between short and long ranges was larger for children than for adults, suggesting higher variability for long distance estimations and accentuated biases for long time estimations in children compared to adults.

**Figure 5:**
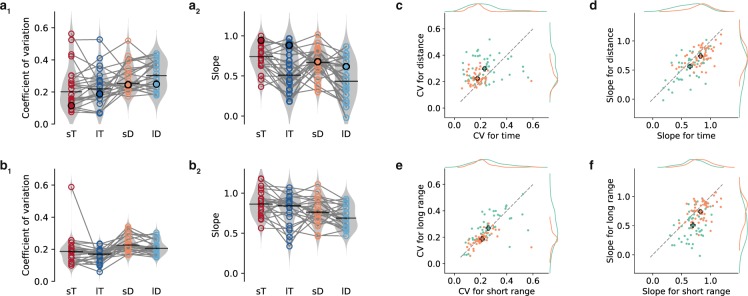
Time and distance estimation performance in adults vs. children. (a&b) Coefficient of variation and slope for short (s) and long (l) ranges in time (T) and distance (D) estimation experiments for all children (**a**) and adults (**b**). Data is displayed similar to Fig. 3. Marker colors identify stimulus range. Filled circles mark the example participant from Fig. 2. (**c**,**e**) Correlations of coefficient of variation (CV) for time vs. distance (**c**) and short vs. long ranges (**e**). Single points correspond to individuals, colors indicate children (green) and adults (orange). Colored plus signs mark averages for adults and children. Gray dashed lines are bisecting lines. Corresponding marginal distributions are given at the right and the top of each plot. (d&f) Correlations of slope for time vs. distance (**d**) and short vs. long ranges (**f**). Similar display as in (c&e).

Finally, we compared performance in time and distance estimation for adults and children. We therefore calculated dimensionless analysis parameters, i.e. coefficient of variation (CV) and slope (Fig. 5a,b), and evaluated differences with three-way rANOVAs. The CV differed significantly for children and adults as well as for stimulus quality, but not for range (children vs. adults: 0.28 ± 0.12 vs. 0.21 ± 0.07, *F*_*1,46*_ = 18.37, *p* = 9 × 10^−5^, *η*^2^ = 0.15; time vs. distance: 0.21 ± 0.11 vs. 0.28 ± 0.08, *F*_*1,46*_ = 16.3, *p* = 2 × 10^−4^, *η*^2^ = 0.11; Fig. 5c,e). Additionally, the interaction of group and stimulus quality was significant (*F*_*1,46*_ = 4.85, *p* = 0.033, *η*^2^ = 0.01). Yet, effect size was small and *t*-tests showed no significant differences between long and short range for neither group (children: *t*_23_ = 1.25, *p* = 0.223; adults: *t*_23_ = 1.89, *p* = 0.071). There were some children with CVs as low as the adults; other children had high CVs. The slopes were significantly different for children and adults but also for stimulus quality and range (three-way rANOVA; children vs. adults: 0.60 ± 0.23 vs 0.79 ± 0.18, *F*_*1,46*_ = 20.95, *p* = 4 × 10^−5^, *η*^2^ = 0.20; time vs. distance: 0.73 ± 0.23 vs. 0.66 ± 0.22, *F*_*1,46*_ = 17.25, *p* = 1 × 10^−4^, *η*^2^ = 0.04; short vs. long: 0.77 ± 0.18 vs. 0.62 ± 0.24, *F*_*1,46*_ = 31.44, *p* = 1 × 10^−6^, *η*^2^ = 0.13; Fig. 5d,f). There was no significant interaction. Again, some children had slopes similar to adults; but there were also children with smaller slopes.

### CV and slope vs. specific skills in children

We also correlated the dimensionless measures with the attention score, with the questionnaire data, and with the task-related questions. For time estimation, CV was decreased with musical experience for short (*ρ* = −0.66, *p* = 4 × 10^−4^) and long range (*ρ* = −0.47, *p* = 0.022), i.e. children who frequently play a musical instrument had fewer variability. Slope correlated positively with musical experience but only for the long range (*ρ* = 0.44, *p* = 0.033). This positive correlation suggests that children who frequently play a musical instrument had less regression in time estimation, at least, for the long range. In addition, there was a correlation of rhythm and CV for time estimation in the long range (*ρ* = −0.417, *p* = 0.043; see Supplementary Table S1 for all correlations).

### Sequential effects

Magnitude estimation is influenced by the order in which stimuli are presented^24^. Models of magnitude estimation that incorporate trial-by-trial updates of internal references or priors by design suggest such sequential effects^28,34,35^. To test for sequential effects, we fitted a generalized linear model (GLM) to the data, comprising both the stimulus in the current trial and in the previous trial as independent variables. Weights for the stimulus in the previous trial were small compared to weights for the stimulus in the current trial (i.e. the slope in the analysis above); Supplementary Fig. S5a. This suggests only little contribution of previous trials. Nevertheless, models without the previous stimulus as independent variable, fit the data less well (Supplementary Fig. S5b). We also fit a GLM that included the previous response instead of the previous stimulus. The distributions of the deviances were negatively skewed, indicating that the previous response even better explains the data than the previous stimulus, however, no case was significant according to likelihood ratio tests (Supplementary Fig. S5c).

### Influence of movement speed

**Figure 6:**
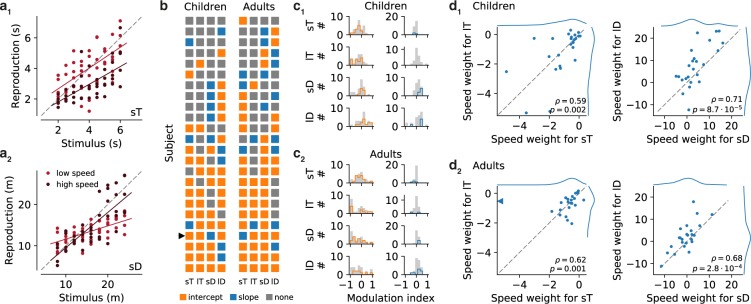
Dependence of time and distance estimation on movement speed. (**a**) Examples of speed-modulation in time (a_1_, short time intervals, sT) and distance estimation (a_2_, short distances, sD) for one child. Individual reproduced values are given as dots. Trials at low speed are indicated by light red and trials at high speed by dark red. Solid lines of matching color are corresponding linear fits. Gray dashed lines mark the bisecting line. The participant displays a significant shift to smaller reproductions at high speed for sT but a significant increase in the slope of the range of reproductions at high speed for sD. (**b**) Characteristics of speed dependence across children and adults. Each square marker indicates a significant shift (intercept change, orange), a slope change (blue) or a none significant effect, respectively, in a particular stimulus range (columns). Each row corresponds to one participant. The arrow head marks the participant from (**a**). (**c**) Histograms of modulation indices for children (c_1_) and adults (c_2_). Left column of panels displays effect sizes for intercept and right panels for slope. Colored outlines indicate participants with significant influence on intercept or slope, respectively. (**d**) GLM analysis of speed dependence. Comparison of weights for speed in the GLMs for sT and lT (left panels), and for sD and lD (right panels) for children (d_1_) and adults (d_2_). Gray dashed lines are bisecting lines. Spearman *ρ* and *p*-values are given in each panel. Marginal distributions are given at the right and the top of each plot. The arrow head in the left panel of d_2_ points to an outlier at (−8.3, −0.5).

We used different movement speeds in the measurement and reproduction phase to uncouple time and space. As a final analysis, we therefore determined how these different speeds influenced time and distance estimation. During measurement, speed was always at 4 m/s; during reproduction, it was between 1.6 and 6.4 m/s (see Methods). We thus split the reproduction data into those acquired at low (1.6–3.6 m/s) and high (4.4–6.4 m/s) reproduction speed. In principle, two different pictures could emerge: Either reproductions could shift to higher/lower values (change in intercept) or the slope of the reproductions could change. We found both scenarios (Fig. 6a). To determine significant changes, we performed ANCOVAs. Speed dependence was significant for 23/24 children and all adults in at least one stimulus range, indicating a general impact of movement speed on time and distance estimation in our task. However, the overall picture was heterogeneous. Only in about half of the participants, speed-dependence was found for at least three stimulus ranges; mostly the intercept was affected (Fig. 6b).

To access speed influence quantitatively, we computed a modulation index for each participant and stimulus range (see Methods). This index scales between −1 (smaller slope or intercept for high speed) and 1 (larger slope or intercept for high speed); Fig. 6c. The distributions were generally broader for the intercept than for the slope. For time estimation in children, significant effects usually were due to a shift to lower reproduced values with higher speed (decrease in intercept). For distance estimation, there was often an increase in intercept. In adults, the picture was less consistent. Significant effects were due to both increases or decreases in intercept. Effects on the slope were less frequent in both children and adults.

In an attempt to resolve this puzzling picture and as an alternative to splitting the data into arbitrary groups (i.e. low and high speed), we included speed as an independent variable into the GLM analysis used above for sequential effects. Speed had a significant impact in most participants (Supplementary Fig. S6a). The emerging picture was similar to the previous analysis but appeared less heterogenous (Fig. 6d). Weights for speed in time reproduction were negative or close to zero for both children and adults, indicating an inverse relation between time reproduction and speed. Weights for individuals strongly correlated for short and long ranges. For distance estimation, weights were mostly positive for both children and adults, and again strongly correlated for short and long ranges. For time estimation, weights appeared more broadly distributed in children; for distance estimation weights for adults were shifted to lower values compared to those of the children. We also included a factor for the speed in the previous trial into the GLM to determine whether a regression effect in estimating speed may influence reproductions. However, the corresponding weights were small compared to those for the current trial’s speed (Supplementary Fig. S6b) and only contributed significantly in very few participants (Supplementary Fig. S6c).

In sum, the movement speed during reproduction affects time and distance reproduction, with larger speed leading to larger reproduced distances but shorter reproduced time intervals.

## Discussion

The aim of the present study was to investigate characteristic biases – namely regression and range effects – in the estimation of the magnitude of temporal intervals and spatial distances. We used an egocentric navigation task (path integration), in which participants had to first estimate and then reproduce either the time that elapsed or the distance they covered while moving through a virtual environment. Participants were eleven-year-old children and young adults. Eleven-year-olds are especially interesting, because they are at the border to adolescence, where cognitive skills are rather mature, but still develop fast. Here this age group served as a starting point for applying the egocentric navigation paradigm in children who are able to meet (on average) the attentional and motivational demands of the task. Further, there have been only few studies examining this age group; especially with respect to distance estimation. In general, regression effects were found for children and adults. Both groups showed stronger regression effects for longer than shorter stimulus ranges and for distance compared to time. Effects were more heterogeneous in children and thus here appeared more pronounced compared to adults. A few children showed veridical time and distance estimation similar to that of adults; the others displayed much stronger regression effects. Finally, individual differences between children in playing a musical instrument were related to time estimation performance.

Regression and range effects as well as scalar variability have been studied in timing experiments with children previously. In line with our results, age-related decreases in the strength of the regression effect have been found for children aged 6 to 14 (up to adulthood) during time reproduction of intervals around 1 s^31^ and for several seconds^36–38^. Note, however, that the last three studies interpreted and discussed their data differently from us. For example, developmental changes in motor responses are suggested as causing the age-related differences. We favor a less rigid interpretation of the mechanisms underlying the regression effect, which may include motor maturation as one of several influences (cf. also Ref.^31^). In contrast to our results, Eisler and Eisler^39^ reported no differences between children aged from 11 to 13 years and adults for reproduction of durations between 1.3 to 20 s. However, their group of 11-to-13-year-old children was slightly older (and, thus, maybe already more mature) than ours and only included 12 children, which might not provide sufficient power for detecting subtle differences between age-groups. Another difference, which is also a problem in a lot of other studies, is that reproduction data is pooled across participants hiding important inter-individual variability. Instead, we first calculated appropriate statistical parameters to describe the data and then pooled those to describe the population.

In addition to changes in the regression effect, we found age-related reductions in the variability of time estimations as has been reported before by others^18,38,40–43^.

Comparatively little is known on distance estimation or in general on path integration in children. Like for timing tasks, accuracy is usually lower in path integration experiments in children aged around four to seven years^14^ for triangle completion^44,45^, and distance estimation^46^ (but see Ref.^47^ reporting less variation in children compared to adults in a condition including both visual and self-motion information). In our experiments, distance estimation was less accurate and regression effects were stronger in eleven-year-olds compared to adults. In general, coefficients of variation and regression slopes were heterogeneous across the group of children, with some being similar to adults and others exceeding the values found for adults.

Sciutti *et al*.^32^ examined regression and range effects for the reproduction of lengths in children aged 7 to 14 and adults. Both groups showed similar regression effects, but younger children showed less accuracy (bias) and less precision (coefficient of variation) that improved with age. These findings are in line with ours. In contrast to Sciutti *et al*.^32^, we found that regression effects became smaller with age. This may be due to the fact that our participants used spatial lengths in the range of several meters instead of centimeters and that our participants reproduced (virtual) whole-body movements instead of moving only a finger. Despite testing for two partly overlapping ranges of stimuli, Sciutti *et al*.^32^ only showed visual evidence for range effects but did not analyze their data accordingly.

A further aim of our study was the comparison of time and distance estimation in the very same behavioral paradigm. Only few studies with children compared estimation of different magnitudes in one setting. Droit-Volet *et al*.^48^ tested time, number and length discrimination in a bisection task in children aged between five and eight. They found that Weber ratios were comparable between the domains, but only if all tasks were presented in sequential order. Otherwise, the Weber ratio of time was larger, indicating lower performance, obviously due to the sequential nature of time.

In our experiments, using movements (egocentric navigation) in virtual reality as stimulation and read-out, we think that we provided most similar conditions for both time and distance estimation, and recorded more ecological data. In the dimensionless measures – coefficient of variation and slope – both groups performed better in estimations of time than of distance. However, we did not observe any reliable interaction between modality (i.e. time or distance) and range. The outcome of the dimensional measures gave hints that children had a higher variability for long distance estimations and accentuated biases for long time estimations compared to adults. Martin *et al*.^49^ found impacts of duration on the estimation of surface and of numerosity in experiments testing all three magnitudes in one experiment. However, duration estimation was unaffected by surface and numerosity. They conclude that their results question the existence of one simple universal magnitude system^50^. Since our study was not designed to tackle such a question, our results do not speak for or against a universal magnitude system. Our analysis comparing actual and calculated reproductions from counterpart time and distance sessions, at least suggests a mutual influence of time and distance. In particular, distance estimation seems to be affected by the passage of time, which is in line with the findings by Martin *et al*.^49^. This notion, however, deserves further investigation.

We used different movement speeds in measurement and reproduction to uncouple time and space, respectively. During time estimation, reproductions were often smaller with higher speed. For distance estimation, the picture was reversed. Both results are compatible with a drift-diffusion model^34^ where the movement speed from our experiments could influence the internal processing speed (drift rate or speed of evidence accumulation over time). Faster processing (integration over time) leads to underestimations (lower reproduced values) for time and overestimations for distance. This view is in line with the results of Martin *et al*.^49^, who report that reducing the speed of stimulus presentation lead to underestimations of surface area.

Recently, regression and range effects have been suggested to result from error reduction strategies implemented by the brain to cope with uncertainty about sensory information by including prior knowledge about the stimulus^24^. A fusion between immediate information about the stimulus and prior knowledge is inherent in Bayesian models^28,30^. A concept that shares remarkable similarities with the internal reference model proposed by others^35^. Bayesian models^21^ but also approaches like drift-diffusion^34^ showed that the regression effect may be a way to minimize errors in magnitude processing that result from both variability of the physical stimuli to be estimated and the individual’s sensory acuity. Finding regression effects in children, the present work implies that error minimization capabilities are present in children. The larger variability we found in some children’s responses could particularly benefit from such error minimization. Alternatively, the lower performance of some children might be explained in terms of less accurate estimation in these individuals^21,32,34^. Nevertheless, future research is necessary to establish at greater detail, how error minimization strategies change during adolescence.

Experience with making music was related to fewer variation and less regression in time estimation in our children as already reported for adults^21–23^. This is interesting, because our children were far from being professional musicians as in most of the studies with adults. One major problem of studies trying to relate musical experiences with timing estimation is the impossibility to disentangle innate music ability and effects of training. Either one of these factors or both (or even third variables) could contribute to superior time processing in musicians, even when tested in the visual modality. In our study, children who played a musical instrument had only few years of training (mean: 3.8, STD 1.1, range 2–5 years) and, nonetheless, they showed improved time estimations. It would be interesting to know whether experience with specific musical instruments has an effect on time estimation in children as shown, e.g., for adult percussionists^21^. However, since only half of our children played a musical instrument regularly and since they played different instruments, we have to leave such questions open for further investigation. Research including longitudinal training studies may shed light onto a link between (specific) musical experience and temporal processing. Interestingly, Agrillo and Piffer^22^ have shown that musicians also outperform non-musicians in spatial discrimination. In contrast to this, we found no correlations of playing a musical instrument and distance estimation in children.

Other subjective measures helped in estimating time as well: while applying a strategy led to reduced bias in short range time estimation, applying a rhythm led to reduced bias, RMSE and CV in long range time estimation. However, these results have to be interpreted with caution, as we used subjective measures in the questionnaires and, in fact, children might have applied different kinds of strategies.

Finally, we did neither find a correlation of attention with time estimation nor with distance estimation. This is in contrast to studies that observed effects of attention on time estimation in children^18,41,51–53^ but might be due to different tasks and different tests for measuring attentional influence. Alternatively, as our children were older than the children in the former studies, it might be that their attention is more mature and hence similar to that of adults affecting time estimation less than in younger children.

## Methods

### Participants

A group of 24 children were tested (12 males). All children were 5^th^ graders (mean age: 11 years, range: 10–12 years) and were recruited from a local gymnasium. Informed consent was obtained from the children and their parents. According to a questionnaire all children had normal or corrected-to-normal vision and none of the children reported attentional problems. All children had average or above-average scores in the d2-R [33; mean percentile rank 55; range: 16–100; 33], a test which measures attention. We asked the parents to indicate the amount of time their children spent doing sports, playing computer games, or playing a musical instrument on a scale between 0 and 4 (0-never; 1-rarely, 1–3 times a month; 2-sometimes, once a week; 3-regularly, several times a week; 4-daily). Twelve children played a musical instrument regularly (including various instruments like piano, horn, euphonium, guitar, violin, harp, flute and German flute). Even three of the children played more than one musical instrument. We asked children to indicate whether they used some kind of rhythm or another strategy for task solving, and whether they did not try to reproduce the time or the distance to the best of their abilities. Here, the scale ranged between 0 and 4 (0-no; 1-yes, but rarely; 2-yes, sometimes; 3-yes, often; 4-always). Children received a voucher of a local toy store or bookshop in appreciation for their participation. A group of 24 young adults (mean age: 22, range: 20–27 years; 15 females) was tested in identical experiments. All had normal or corrected-to-normal vision and none reported attentional problems.

The study was approved by the ethical committee of the German Psychological Association (“Ethikkommission der Deutschen Gesellschaft für Psychologie”, US042015) in accordance with the Declaration of Helsinki.

### Procedure

The experiment was embedded in a child-friendly cover story. All participants were told they were an astronaut lost on a foreign planet where they had to search for puzzles in four missions to return to Earth. Each participant completed four missions in two sessions on two different days. In the first session, participants received two missions (one for time, one for distance) with short stimulus distributions (see below), each consisting of 90 trials. In the second session participants received two missions with long stimuli also consisting of 90 trials each. Every session lasted approximately 60–70 minutes. Between the two missions of a session, participants were allowed to take a break of at least five minutes. In addition, children completed the d2-R-attention test^33^ during the break in the first session. The d2-R is a classical paper-pencil attention test. Each row contains a mixture of printed letters: *d*s and *p*s with one to four short lines above and/or below each single letter. Participants have to cancel out the target letter d containing exactly two lines without making errors or missing a target. For each row of printed letters, the time limit is 20 seconds. The whole test lasts approximately 10 minutes. The score of attention integrates the number of correctly identified targets, false negative and false positive errors.

Before each mission, participants received detailed instructions about the upcoming mission and their attention was directed to the fact that they should base their estimates solely on either the time or the space dimension. For time estimation missions, participants were explicitly instructed not to count as recommended by Rattat and Droit-Volet^54^.

Participants were comfortably seated in front of a computer screen (distance to the screen was approximately 50 cm). The virtual reality environment was presented with Vizard v5 (WorldViz, http://www.worldviz.com) and depicted a stone desert. As in Petzschner and Glasauer^28^ the orientation of the ground texture, the position of the stones and the starting position were randomized in each trial. The eye height in the virtual reality was adjusted to the individual eye height of each participant^55^. Figure 1a depicts the sequence of events in a trial. In the measurement phase, participants initiated virtual visual self-movement by pressing the space bar of the PC’s keyboard with the index finger of their dominant hand. They were instructed to keep pressing the key until the movement stopped automatically. After that, they initiated the reproduction phase by pressing the space bar again. This time they were instructed to keep pressing the space bar for as long as they had experienced the time or the distance of movement in the measurement phase. A symbol in the upper left corner reminded participants whether they were in the measurement or reproduction phase (Fig. 1a). After each trial, a piece of a puzzle was collected, independent of performance. Each mission finished when the mission’s puzzle was complete (90 trials), solving the cover story.

There were ten practice trials at the beginning of each mission. Here, but not in the actual experiment, participants received positive feedback if they hit a rather wide window of +/− 50% of the stimulus, and “encouragement” to try better otherwise.

### Stimuli

The order of time and distance estimation within each session was balanced between participants, resulting in four possible sequences (td-TD, td-DT, dt-DT, dt-TD; t - short time estimation, d - short distance estimation, T - long time estimation, D - long distance estimation). For each mission, nine different stimuli were chosen from uniform sample distributions (short time: 2, 2.5, 3, 3.5, 4, 4.5, 5, 5.5, 6; short distance: 8, 10, 12, 14, 16, 18, 20, 22, 24; long time: 4.5, 5, 5.5, 6, 6.5, 7, 7.5, 8, 8.5; long distance: 18, 20, 22, 24, 26, 28, 30, 32, 34; see also Fig. 1b). Short and long distributions overlapped in four values (underlined). From the sample distributions 90 trials were randomly drawn such that each single stimulus was presented 10 times within each mission. Appropriate randomization of the stimulus sequences was ensured beforehand by testing (χ^2^ test) for local uniform distribution in sliding windows with a width of nine consecutive stimuli.

During measurement, movement speed was set to 4 m/s. For reproduction, this speed was multiplied by a gain factor that was chosen from a uniform distribution ranging between 0.4 and 1.6 binned into 10 equally-spaced values (gain factors of one were excluded since they were used during measurement), resulting in movement speeds between 1.6 and 6.4 m/s. Speed was thus different between measurement and reproduction phases; and movement time and distance were disentangled, excluding the respective alternative solution strategy. We created the stimulus sequences beforehand and ensured that each gain was equally often presented for each stimulus so that no correlations were introduced with the stimulus values.

### Data analysis

Data analysis was done with Python v2.7 using the packages Numpy v1.11, Scipy v0.19, Statsmodels v0.8, and Matplotlib v2.0. For statistical testing we also used R (v1.1.414). We excluded reproductions shorter than 0.5 s for time estimations or one meter for distance estimations, or longer than 60 seconds or 100 meters, which were due to slips from the keyboard’s space bar or mixing up measurement and reproduction phase, respectively (children: 1.05% of all trials; adults: 1.12% of all trials).

To compare the results across participants, we extracted a couple of different parameters; see also Fig. 2b. The mean squared error MSE measures the deviation between stimulus *s* and response *r* (i.e. reproduction) ${\rm{MSE}}(r)={\rm{E}}[{(r-s)}^{2}]$. The MSE can be split into two contributions

$$\begin{array}{rcl}{\rm{MSE}}(r) & = & {\rm{Var}}(r)+{{\rm{BIAS}}}^{2}(r)\\  & = & {{\rm{E}}}_{s}[{{\rm{Var}}}_{s}(r)]+{{\rm{E}}}_{s}[{{\rm{BIAS}}}_{s}^{2}(r)]\\  & = & {{\rm{E}}}_{s}[{{\rm{E}}}_{r}[{(r-{{\rm{E}}}_{r}[r|s])}^{2}|s]]+{{\rm{E}}}_{s}[{({{\rm{E}}}_{r}[r|s]-s)}^{2}]\end{array}$$

where Var_*s*_(*r*) and BIAS_*s*_^2^(*r*) are the variance and squared bias, respectively, of the responses for stimulus *s*, and *E*_*s*_[.] and *E*_*r*_[.] denote expected values over stimuli *s* and responses *r*, respectively. Partitioning the MSE as above, we can separate the general variability in the responses Var(*r*) from systematic biases BIAS^2^(*r*). Note that we report the square roots of the above parameters, i.e. standard deviation STD, $\sqrt{{{\rm{BIAS}}}^{2}}$ and RMSE, to provide values in units of the estimated quantity.

Since $\sqrt{{{\rm{BIAS}}}^{2}}$ does not contain information about the direction of systematic errors, like general under- or overestimation, we also calculated the $BIAS(r)={E}_{s}[{E}_{r}[r|{\rm{s}}]-s]$.

For comparing time and distance estimation data, we computed (1) the coefficient of variation for the responses *r* as the average coefficient of variation for each stimulus in one stimulus range, i.e., $CV={E}_{s}[\frac{{\rm{STD}}(r|{\rm{s}})}{{E}_{r}[r|{\rm{s}}]}]$ and (2) the slope of the linear regression between stimulus and response (i.e. reproduction). A slope of one would correspond to veridical estimation (i.e., no regression) and smaller slopes to stronger regression.

For the paired data in Figures 3 and 4, we first tested the individual data sets for normality with Shapiro-Wilk tests. In most cases, at least one of the compared data sets was significantly different from normality. Therefore, we decided to use non-parametric Wilcoxon signed-rank tests. To compare data from children with that of adults, we used two-way or three-way mixed repeated-measures ANOVAs (Figs. 4 and 5), due to lack of appropriate non-parametric tests. Significant modulation of time and distance reproduction by movement speed was determined with analysis of covariance (ANCOVA). Modulation indices were calculated as (*x*__high_ − *x*__low_)/(|*x*__high_| + |*x*__low_|) with *x* being either the slope or the intercept of the linear fit between stimuli and reproduced values at high and low speed, respectively. Finally, we calculated Spearman correlations between all measures of time and distance estimations and questionnaire data.

### Generalized linear model (GLM) analysis

We fitted various GLMs comprising different numbers of independent variables and assuming normally distributed responses as dependent variable: (1) To test for sequential effects we used

$${r}_{i}={a}_{1}{s}_{i}+{a}_{2}{s}_{i-1},$$

where *r*_*i*_ is the response in the *i*th trial, *s*_*i*_ is the stimulus in the same trial and *s*_*i-1*_ is the stimulus in the previous trial. Alternatively, we included the previous response *r*_*i-1*_ instead of the previous stimulus *s*_*i-1*_. (2) When testing for speed effects, we extended the model by two independent variables for the speed *v*_*i*_ in the current and *v*_*i-1*_ in the previous trials, resulting in the model.

$${r}_{i}={a}_{1}{s}_{i}+{a}_{2}{s}_{i-1}+{a}_{3}{v}_{i}+{a}_{4}{v}_{i-1}.$$

The models were fitted using iteratively reweighted least squares from Statsmodels’ GLM implementation. Deviance between two models was calculated as the difference between their log-likelihoods multiplied by two and was tested for significance with a likelihood-ratio test.

## Electronic supplementary material

SUPPLEMENTARY INFORMATION

## Data Availability

The datasets generated during and/or analyzed during the current study are available from the authors on reasonable request.
