# Supplementary material for: Time and distance estimation in children using an egocentric navigation task
Source: Sci Rep. 2018 Dec 20;8:18001. doi: 10.1038/s41598-018-36234-1 (PMC6302095; doi:10.1038/s41598-018-36234-1)
Supplement: Supplementary file 1 — SUPPLEMENTARY INFORMATION [file 41598_2018_36234_MOESM1_ESM.pdf]

Supplementary information

Time and distance estimation in children using an egocentric navigation task

Kay Thurley & Ulrike Schild

Tables

S1 Correlations between time and distance estimation and questionnaire data 2

Figures

S1 Example for comparing responses from time and distance estimation 3
S2 Comparison of time and distance estimation experiments in children 4
S3 Comparison of time and distance estimation experiments in adults 5
S4 Correlation of responses during time and distance estimation 6
S5 Influence of stimulus sequence 7
S6 Influence of movement speed 8

Table S1. Spearman correlations between all measures of time and distance estimation and questionnaire data. Significant correlations ($\alpha < 0.05$) are shaded in grey.

	Attention test (d2-R)	Effort/ subjective attention during the task	Playing a musical instrument	Sport activity	Playing computer games	Using a strategy in estimation	Using rhythm during the task
Time							
sT_squared bias	*0.205<br>-0.268	0.095<br>0.349	0.027<br>-0.451	0.713<br>-0.079	0.367<br>-0.193	0.102<br>0.342	0.452<br>-0.161
sT_STD	0.266<br>-0.236	0.864<br>-0.037	0.001<br>-0.647	0.293<br>-0.224	0.303<br>-0.219	0.697<br>0.084	0.073<br>-0.373
sT_ RMSE	0.216<br>-0.262	0.929<br>0.019	0.001<br>-0.635	0.318<br>-0.213	0.263<br>-0.238	0.540<br>0.131	0.149<br>-0.303
sT_bias	0.989<br>-0.003	0.698<br>-0.084	0.920<br>0.022	0.331<br>0.207	0.125<br>0.322	0.025<br>-0.457	0.685<br>0.087
sT_CV	0.260<br>-0.239	0.888<br>-0.030	<0.001<br>-0.663	0.242<br>-0.248	0.202<br>-0.270	0.572<br>0.121	0.063<br>-0.386
sT_slope	0.388<br>0.184	0.099<br>-0.345	0.067<br>0.380	0.757<br>-0.067	0.054<br>0.398	0.051<br>-0.403	0.316<br>0.214
lT_ squared bias	0.694<br>-0.085	0.304<br>0.219	0.170<br>-0.290	0.776<br>-0.061	0.542<br>0.131	0.165<br>0.293	0.026<br>-0.452
lT_STD	0.653<br>-0.097	0.929<br>0.019	0.013<br>-0.500	0.298<br>-0.222	0.719<br>-0.078	0.654<br>0.096	0.244<br>-0.247
lT_RMSE	0.899<br>-0.027	0.858<br>0.038	0.038<br>-0.425	0.791<br>-0.057	0.590<br>-0.116	0.359<br>0.196	0.042<br>-0.419
lT_bias	0.526<br>-0.136	0.950<br>0.013	0.934<br>0.018	0.180<br>-0.283	0.862<br>-0.037	0.324<br>-0.210	0.158<br>0.297
lT_CV	0.926<br>-0.020	0.853<br>0.040	0.022<br>-0.465	0.752<br>-0.068	0.497<br>-0.146	0.349<br>0.200	0.043<br>-0.417
lT_slope	0.648<br>0.098	0.550<br>-0.128	0.033<br>0.436	0.327<br>0.209	0.828<br>-0.047	0.067<br>-0.379	0.061<br>0.388
Distance							
sD_squared bias	0.563<br>0.124	0.159<br>0.297	0.808<br>-0.052	0.563<br>0.124	0.557<br>0.126	0.730<br>0.074	0.258<br>0.241
sD_STD	0.140<br>-0.310	0.407<br>-0.177	0.629<br>-0.104	0.449<br>-0.162	0.947<br>0.014	0.105<br>-0.339	0.162<br>-0.295
sD_RMSE	0.514<br>-0.140	0.887<br>0.031	0.686<br>-0.087	0.446<br>-0.163	0.716<br>0.078	0.414<br>-0.175	0.500<br>-0.145
sD_bias	0.222<br>-0.259	0.477<br>-0.152	0.295<br>-0.223	0.183<br>0.281	0.997<br>0.001	0.064<br>-0.383	0.304<br>-0.219
sD_CV	0.320<br>-0.212	0.867<br>-0.036	0.752<br>-0.068	0.073<br>-0.373	0.664<br>0.094	0.277<br>-0.231	0.245<br>-0.247
sD_slope	0.529<br>-0.135	0.303<br>-0.219	0.788<br>0.058	0.720<br>0.077	0.446<br>0.163	0.069<br>-0.377	0.459<br>-0.159
lD_squared bias	0.974<br>0.007	0.172<br>0.288	0.480<br>-0.151	0.839<br>-0.044	0.774<br>0.062	0.079<br>0.365	0.840<br>0.044
lD_STD	0.221<br>-0.259	0.593<br>0.115	0.846<br>-0.042	0.114<br>-0.331	0.383<br>0.186	0.414<br>-0.175	0.497<br>0.146
lD_RMSE	0.194<br>-0.275	0.290<br>0.225	0.755<br>-0.067	0.351<br>-0.199	0.251<br>0.244	0.814<br>0.051	0.435<br>0.167
lD_bias	0.49<br>-0.147	0.792<br>-0.057	0.940<br>0.016	0.639<br>0.101	0.458<br>0.159	0.064<br>-0.384	0.838<br>-0.044
lD_CV	0.234<br>-0.252	0.590<br>0.116	0.838<br>-0.044	0.075<br>-0.371	0.535<br>0.133	0.825<br>-0.048	0.512<br>0.141
lD_slope	0.926<br>-0.020	0.427<br>-0.170	0.229<br>0.255	0.687<br>-0.087	0.451<br>-0.161	0.097<br>-0.346	0.815<br>0.050

* *p*-values are displayed above *ρ*-values

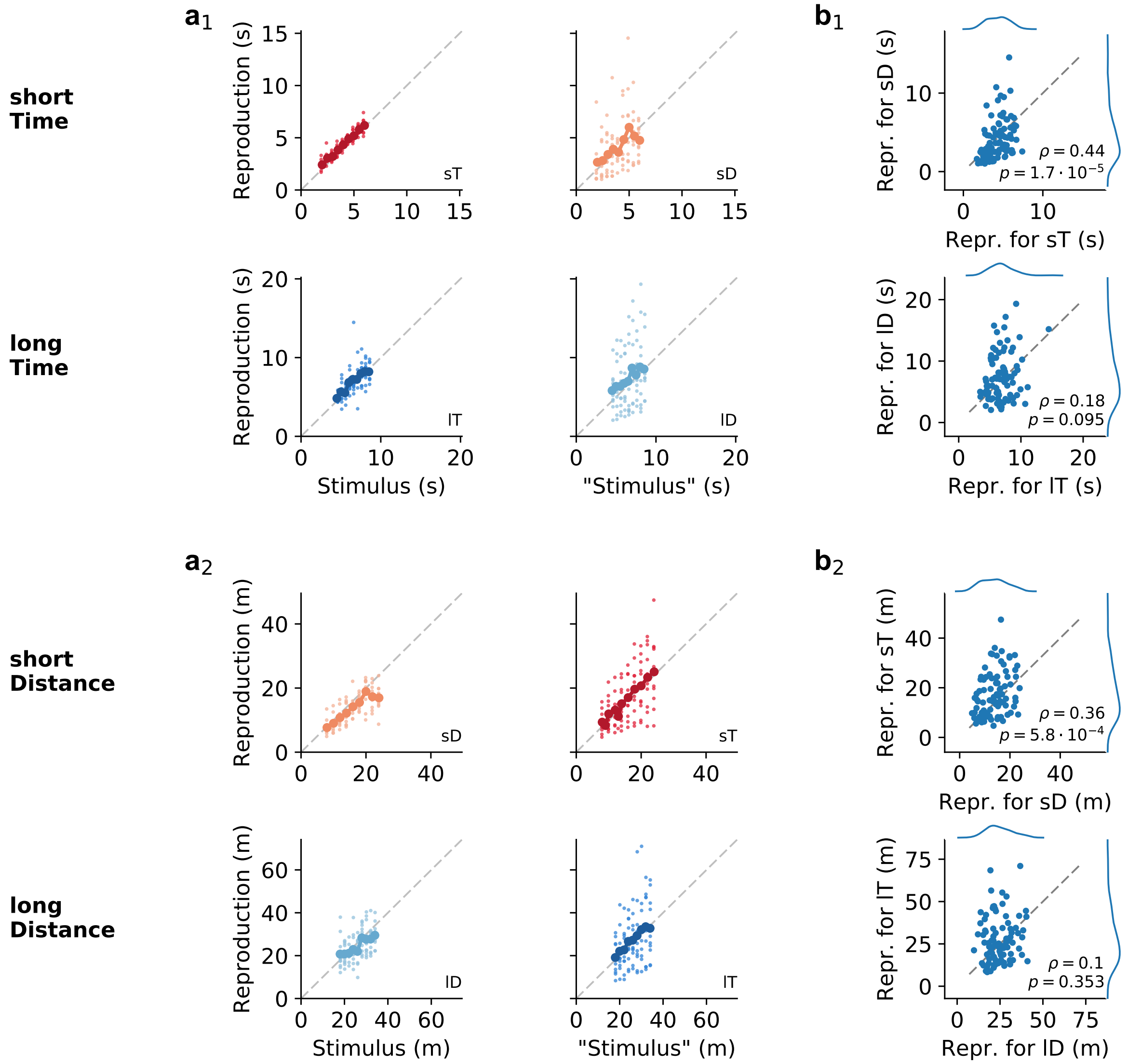

Figure S1. Example for comparing responses from time and distance estimation. Data for the example child from Fig. 2 in the main text is used. (a_1) In the left columns, time reproductions are plotted for short or long stimulus ranges as in Fig. 2a. The right panels show "time" reproductions calculated from the short/long distance experiments by dividing with the speed in each trial. Individual trials are given as small dots and averages for each stimulus as large dots connected by a line. Gray dashed lines mark the bisecting lines. Labels in each panel's lower right corner and colors identify stimulus distributions (cf. Fig. 1b; short time, sT; long time, lT; short distance, sD; and long distance, sD). (b_1) Individual short/long time reproductions and calculated reproductions from the corresponding distance experiments. Gray dashed lines are bisecting lines. Spearman ρ and p values are given in each panel. Marginal distributions are provided at the right and the top of each plot. (a_2&b_2) Similar plots for distance reproduction. Here calculated reproductions are determined by multiplying with each trial's speed.

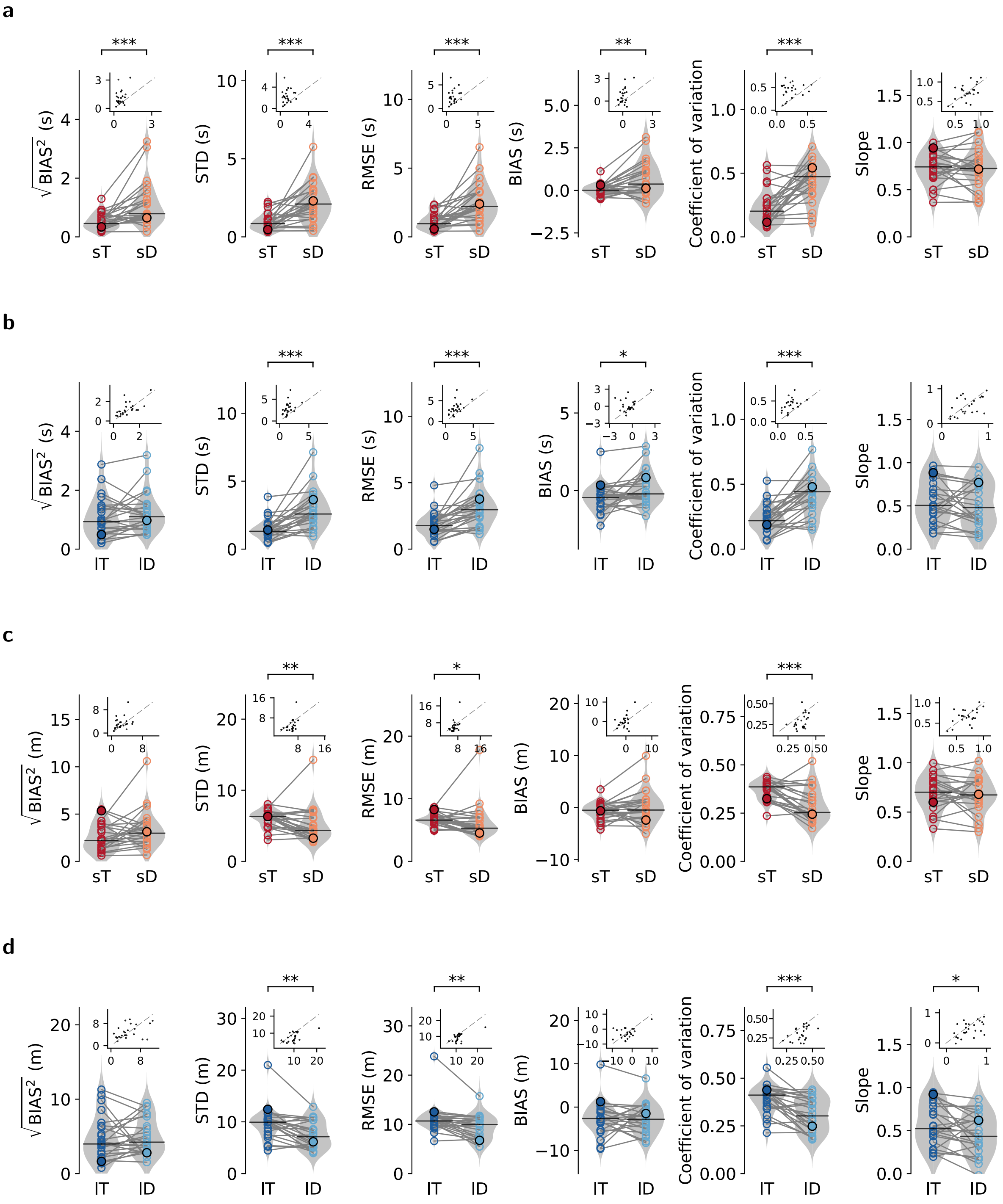

Figure S2. Comparison of time and distance estimation experiments in children. (a&b) Analysis parameters for time experiments (left column in each panel) and for corresponding "time" responses calculated from the distance experiments (right column in each panel) by dividing with the speed in each trial. (c&d) Analysis parameters for distance experiments (right column in each panel) and for corresponding "distance" responses calculated from the time experiments (left column in each panel) by multiplying with the speed in each trial. (a) Short time (sT), (b) long time (lT), (c) short distance (sD), and (d) long distance (sD) experiments. Data of individuals is displayed as open circles connected by a gray line. Filled circles belong to the example participant from Fig. S1. Violin plots illustrate the distribution of the population. A black solid line marks the median. Stars indicate significant difference (Wilcoxon signed-rank tests) with $p \leq 0.05$, *; $p \leq 0.01$, **; $p \leq 0.001$, ***. Inset: Same data as in the main panel but plotting short vs. long for each participant (black dots). Gray dashed lines mark the bisecting lines.

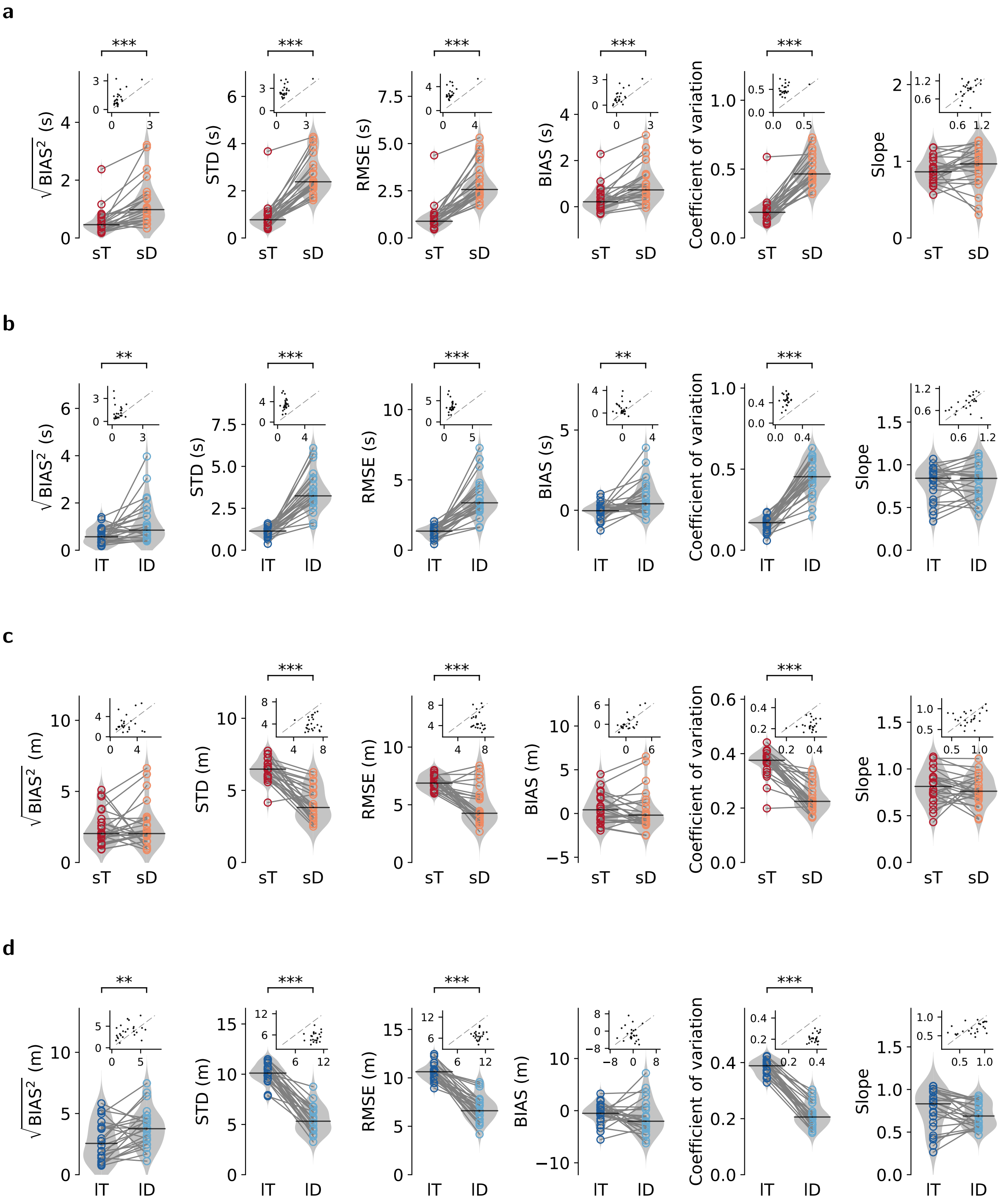

Figure S3. Comparison of time and distance estimation experiments in adults. (a&b) Analysis parameters for time experiments (left column in each panel) and corresponding "time" responses calculated from the distance experiments (right column in each panel) by dividing with the speed in each trial. (c&d) Analysis parameters for distance experiments (right column in each panel) and corresponding "distance" responses calculated from the time experiments (left column in each panel) by multiplying with the speed in each trial. Data is displayed like in Fig. S2.

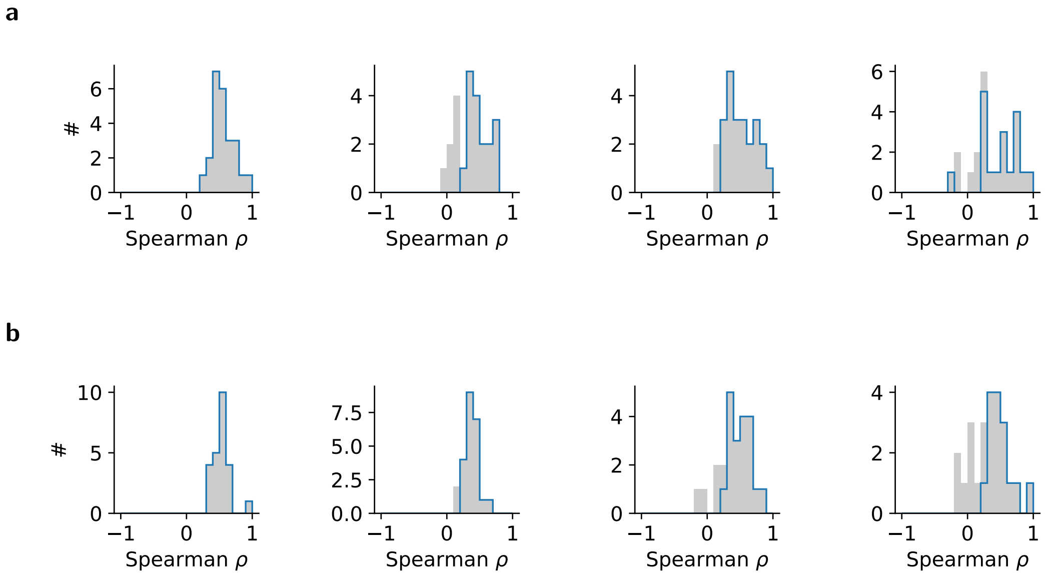

Figure S4. Correlation of responses during time and distance estimation. Correlations of short/long time or distance reproductions and calculated reproductions from the corresponding distance or time experiments (cf. Fig. S1b). Gray bars are Spearman correlation coefficients. Blue solid lines demarcate significant coefficients. (a) Correlations for children and (b) for adults.

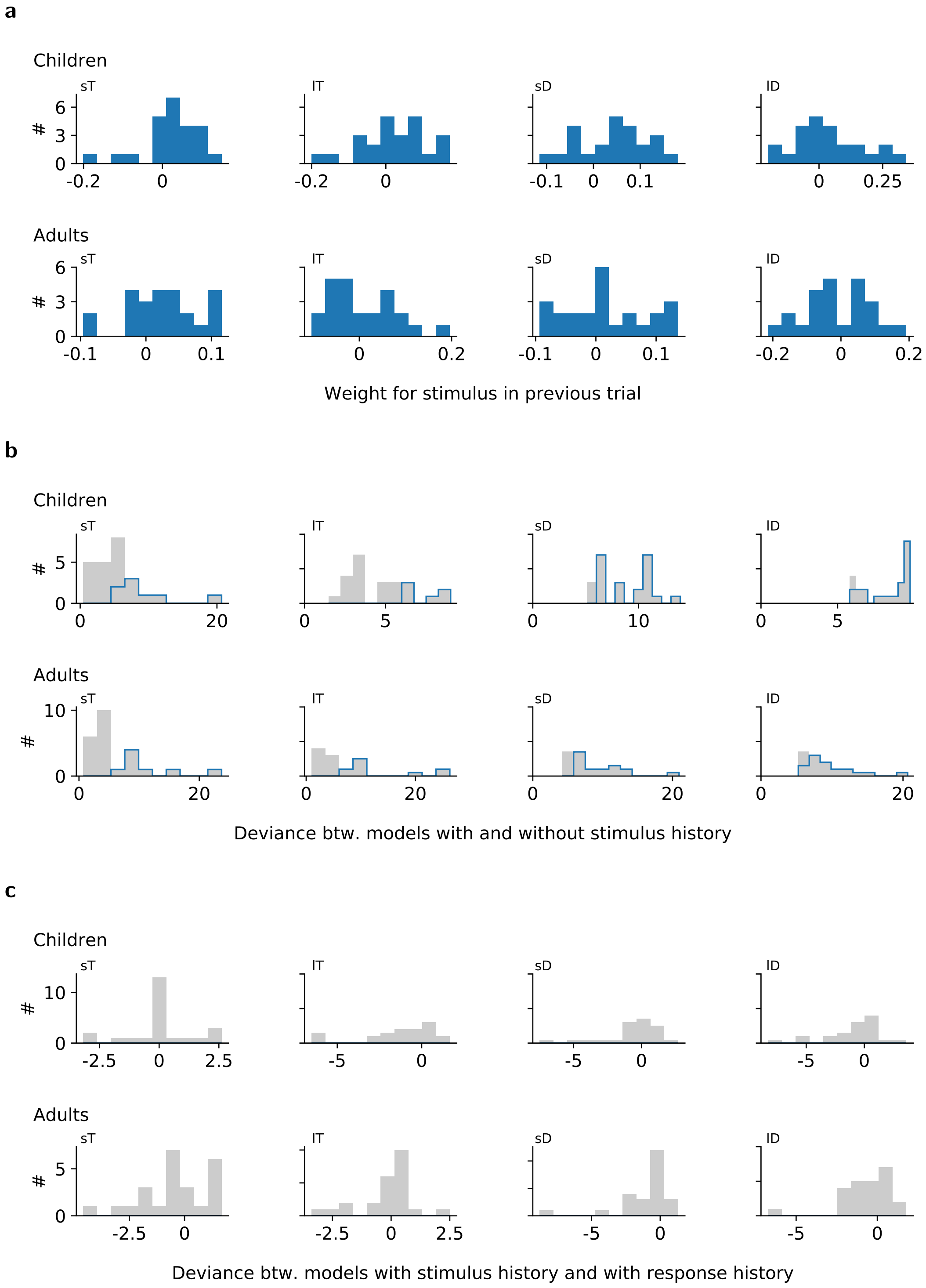

Figure S5. Influence of stimulus sequence. (a) Histograms of GLM weights for stimulus in previous trial in each range (sT, lT, sD and lD) for children (*upper panels*) and adults (*lower panels*). (b) Deviance between GLM models including current stimulus and previous stimulus or only current stimulus as independent variables. Again, histograms are provided for each range (sT, lT, sD and lD) and separately for children and adults. Positive values indicate that stimulus history contributes to the responses. Cases with significant contributions (significant likelihood ratio test) are shown as blue solid lines. (c) Deviance between GLM models including current stimulus, and previous stimulus or previous response as independent variables. The histograms are skewed to negative values, indicating that the previous response may better explain the data than the previous response. However, note that likelihood ratio tests of the deviances were not significant for any range and participant group.

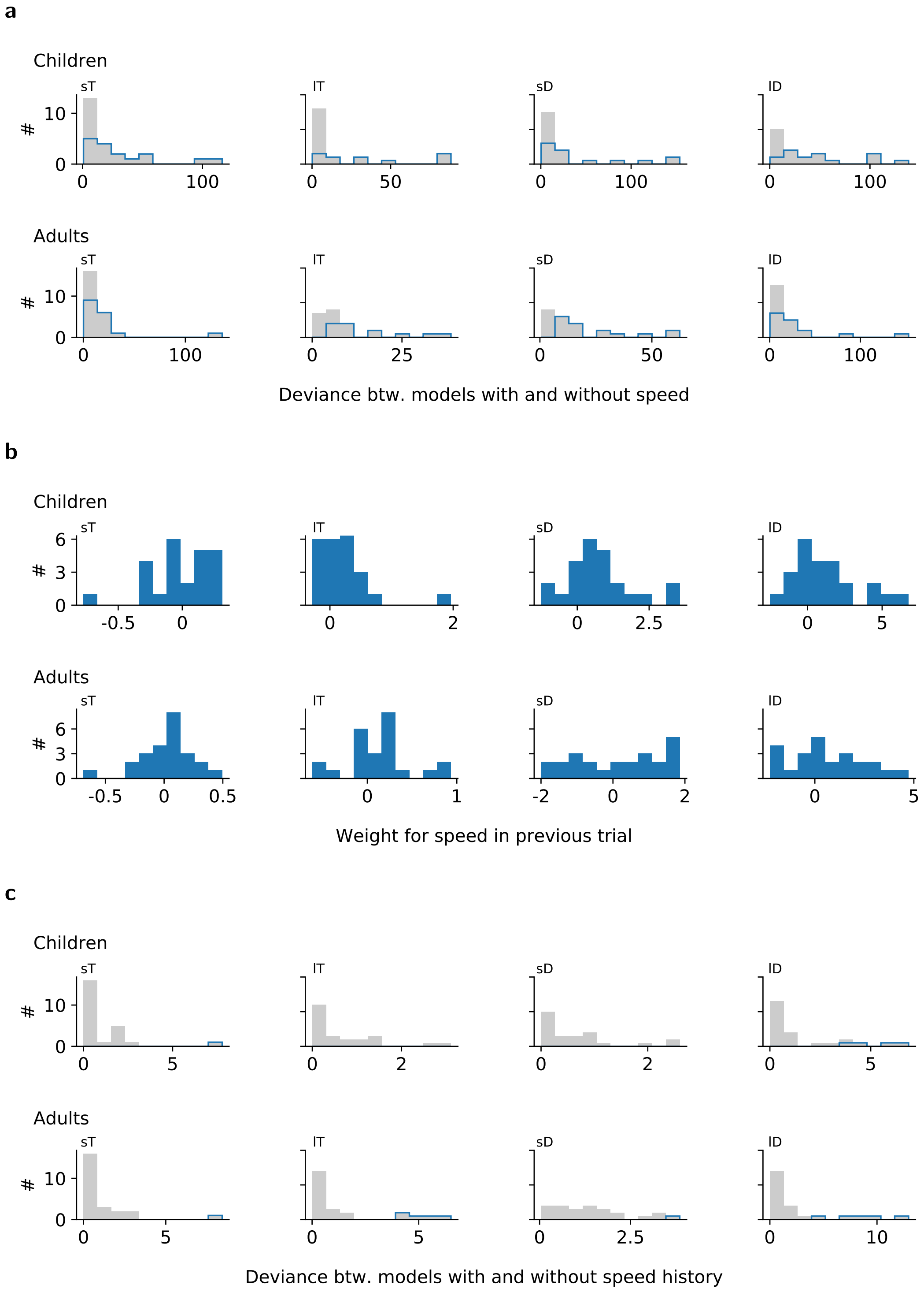

Figure S6. Influence of movement speed. (a) Deviance between GLM models with or without speed as an independent variable. Histograms are provided for each range (sT, lT, sD and lD) and separately for children and adults. Positive values indicate that movement speed contributes to the responses. Cases with significant contributions (significant likelihood ratio test) are shown as blue solid lines. (b) Histograms of GLM weights for speed in the previous trial in each range (sT, lT, sD and lD) separated for children (*upper panels*) and adults (*lower panels*). (c) Deviance between GLM models with or without speed in previous trial as an independent variable. Display as in (a). Positive values indicate that the speed in the previous trial contributes to the responses but only few cases are significant (likelihood ratio test).
